# Theorising the impact of macroturbulence on work and HRM: COVID‐19 and the abrupt shift to enforced homeworking

**DOI:** 10.1111/1748-8583.12465

**Published:** 2022-08-13

**Authors:** Emma Hughes, Rory Donnelly

**Affiliations:** ^1^ University of Liverpool Management School Liverpool UK

**Keywords:** boundary theory, homeworking, social exchange theory, work‐life balance

## Abstract

This paper is among the first to fuse Social Exchange Theory (SET) with Boundary Theory (BT) to expand the knowledge of HR scholars and practitioners on the repercussions of macroturbulence for the management and experience of work. In‐depth interviews were conducted with 102 academics from UK universities to examine the nexus between COVID‐19 and changes to work at meso and micro levels. The findings extend SET and BT by elucidating how complex internal and external social exchange relationships interact more intensely and provoke tensions with a wider array of work‐life boundaries during a profound global crisis. Based on these findings, we advance a new analytical framework which provides a deeper and more integrated theorisation of the interrelationship between macroturbulence and changing work‐life boundaries. Moreover, we identify implications for practice, which have widespread and ongoing significance given that different types of macro‐level change will continue to disrupt working lives.

**Abbreviations**
BT: Boundary Theory
SET: Social Exchange Theory

1

**Practitioner notes**

**What is currently known?**

- Human resources engage in social exchange relationships underpinned by mutual expectations and obligations.
- Macro‐level turbulence and uncertainty can disrupt these social exchanges and adversely affect work‐life balance.

**What this paper adds?**

The paper provides a new multi‐level framework to examine how staff experiences of macro‐level crises are shaped by interactions and tensions between:

- A range of different boundaries (e.g., work‐life, progression, regulatory, control)
- Complex social exchange relationships between parties (e.g., family members, line‐managers, and peers)

**Implications for HR practitioners and organisations**

- Generic well‐being initiatives need supplementing to meet the needs of individual employees, temporary workers, and line‐managers, particularly during macro‐level turbulence.
- Addressing pre‐existing tensions (e.g., over work‐life balance) within social exchange relationships is likely to improve responses to future macroturbulence.
- Greater staff involvement in decisions concerning changing working practices should be encouraged to help navigate future crises.

## 2 INTRODUCTION

Interest in the role of macro‐level turbulence in shaping working conditions and work‐life balance is growing, particularly in the wake of recent (inter)national economic crises (Chatrakul Na Ayudhya et al., 2019; Prouska & Psychogios, 2018; Prouska et al., 2016). Such crises can intensify pre‐existing tensions in socioeconomic relationships and alter work boundaries and conditions, presenting acute challenges for HR scholars and practitioners to address (Hodder, 2020). As new crises continue to emerge and evolve, we need to extend our knowledge of how different types of macro‐level crises influence perceptions and experiences of work and management responses. Thus, we need to move beyond a mono‐theoretical lens and adopt a multilevel analytical framework (Ererdi et al., 2020). To our knowledge, this paper is among the first to fuse social exchange theory (SET) with boundary theory (BT) to provide original and significant extensions to the theorisation of macroturbulence and its impact on work and Human Resource Management (HRM).

We focus on the severe turbulence caused by COVID‐19 because it precipitated unprecedented change and uncertainty for organisations, HRM and staff (Einwiller et al., 2021; Hodder, 2020). Distinctive features of the crisis have included enforced homeworking for prolonged periods, the disruption of conventional work‐life boundaries and the physical distancing of social relationships and exchanges (Anderson & Kelliher, 2020). Thus, we investigated the following research questions as these developments unfolded: How does such macroturbulence shape working conditions, the nature of social exchange relationships and associated boundaries? And how do workers make sense of and navigate these multilevel dynamics?

To address these questions, we used rich data from semi‐structured interviews with academics (*n* = 102) because they engage in important social exchanges with internal and external parties at different levels, and the nature of academic labour, HRM policy and practice in universities is subject to ongoing change (Kalfa et al., 2018).

The findings make important contributions to the combined extension of SET and BT to better theorise and accommodate the nexus between macroturbulence and meso‐ and micro‐level work and HRM outcomes. We also identify significant implications for future HRM research and practice as the COVID crisis and new ones develop.

## 3 AN INTEGRATED MULTILEVEL THEORETICAL PRISM FOR ANALYSING MACROTURBULENCE ON WORK AND HRM

In this section, we set out the theoretical framework for this paper. We then explain the rationale for combining SET and BT to provide a multilevel prism for the theorisation and analysis of macroturbulence on work and HRM.

### 3.1 Social exchanges and their boundaries

The core assumption of SET is that in addition to transactional economic exchanges, employment relationships are social exchanges underpinned by mutual expectations and obligations (Cropanzano et al., 2017; Davis & Van der Heijden, 2018). Staff who consider a social exchange relationship with an organisation to be relatively balanced, where organisations provide appropriate social resources and support in return for their labour, are more likely to feel morally obliged to reciprocate through positive and proactive behaviours (Kelliher et al., 2019). In contrast, when they regard social exchanges to be consistently imbalanced and have unmet expectations, this can lead to reduced affective commitment, productivity, trust, and citizenship (Avgoustaki & Bessa, 2019).

Some studies have explicitly applied SET to examine the impact of macroturbulence on social exchange relationships. For example, Huffman et al. (2021) analysed how furlough policies shaped workers' perceptions of the fairness of their social exchange relationship with their employer. During the 2009–2013 economic recession in Greece, Galanaki (2019) found that the benefits employees received as part of their exchange relationship with their employer consolidated their organisational commitment. Meanwhile, Einwiller et al. (2021) concluded from their survey that the clarity and consistency of internal organisational communications influenced workers' perceptions of the social exchange relationship with their employer in Austrian organisations during the pandemic but called for future research to gain more in‐depth and nuanced insights.

Blau's (1964) and Emerson's (1976) sociological articulations of SET acknowledge that different parties engage in social exchange relationships at micro and macro levels, such as family members, friends, colleagues, and members of broader international social networks. Research engaging with SET in the context of macroturbulence has primarily analysed dyadic social relationships between employers and/or managers and the people they employ or manage, in some cases focussing on the psychological contract as a strand of SET (e.g., Huffman et al., 2021). Exceptions would include Nyfoudi et al. (2020) and Prouska et al. (2022) who found that co‐workers engaged in social exchanges to support each other, navigate changing working conditions and mitigate macroturbulence in Cyprus and Greece. Thus, as social exchanges extend beyond dyadic employment relations, particularly when working from home, we adopted SET in this paper because it provides a broader and more inclusive framework than the psychological contract.

Some scholars have argued that work and employment studies have tended to overlook how imbalanced and consistently contested social exchange relationships are shaped by structural, institutional, and social tensions and ambiguities (Cross & Dundon, 2019; Harney, 2019; Jeske & Shultz, 2016; McDonald & Townsend, 2012). These tensions at the micro (individual), meso (organisational) and macro (external) levels can worsen in a crisis, as employers and workers become more mutually dependent. However, even more collaborative exchange relationships continue to be shaped by patterns of control, conflicting interests, discretion, individual agency, and wider structures (Vincent et al., 2020).

The degree to which workers perceive organisational support for work‐life balance to be inadequate can generate antagonism and tensions within social exchange relationships (Kelliher et al., 2019). Research suggests that macroturbulence such as economic crises negatively affect work‐life interfaces (Chatrakul Na Ayudhya et al., 2019; Hofäcker & König, 2013). However, few studies focussing on macroturbulence and SET have engaged with the concept of work‐life balance (Davis & Van der Heijden, 2018).

In this paper, we combine SET with BT to examine work‐life interfaces (Delanoeije et al., 2019; Nippert‐Eng, 1996). This is because BT encourages a more detailed and dynamic examination of social exchanges at work by segmenting ‘work‐life balance’ to focus on shifting physical, temporal, and psychological boundaries between work and life. Most research examining work‐life boundaries has focussed on how individuals engage in ‘boundary work’ to navigate transitions between family roles and (un)paid work (e.g., Carvalho et al., 2021; Choroszewicz & Kay, 2020). However, as Kelliher et al. (2019) and Allen et al. (2021) have affirmed, we need to embrace a broader conceptualisation of life, which extends beyond fulfiling childcare responsibilities and incorporates additional activities and social relations, such as caring for other relatives and socialising.

Boundary theory is rarely integrated with SET in empirical HRM research, particularly when analysing the impact of macroturbulence on work and HRM. Moreover, studies applying BT in the context of macroturbulence such as COVID‐19 have so far been scarce and have focussed mainly on work‐family dynamics (Carvalho et al., 2021) or individual boundary management preferences (Allen et al., 2021). Therefore, our application of SET, which focuses on the nature of social exchange relationships between various parties and how they are shaped by tensions and ambiguities at different levels can help expand BT by revealing how physical, temporal, and psychological work‐life boundaries connect with a broader range of other context‐specific boundaries. The next section explains and justifies the methodology used in this study.

## 4 METHODOLOGY

As argued by scholars who have examined how economic crises affect work and HRM (e.g., Chatrakul Na Ayudhya et al., 2019; Prouska & Psychogios, 2018), a qualitative mode of inquiry adopting an interpretivist epistemology offers the scope to inductively uncover and probe the complexities of worker experiences, emotions, sense‐making and individual actions. A critical epistemological approach (Edwards, 2017) was also adopted to examine the tensions shaping exchange relationships and boundaries at multiple levels.

### 4.1 Sampling and data collection

102 academics (51 females, 51 males) were interviewed using purposive and chain‐referral sampling (see Chatrakul Na Ayudhya et al., 2019). Academics from the researchers' immediate networks outside their university were invited to participate. The sample was then built progressively through chain referrals to potential study participants who met the criteria of being academics employed by a UK university who were working from home due to COVID‐19.

As with other sampling methods, sampling and participation biases are likely to occur. To minimise the potential for these biases, we interviewed a comparatively large sample of participants for a qualitative interview‐based study (Saunders & Townsend, 2016) from different schools and disciplines at different levels of seniority (see Table 1), expressing a spectrum of views. Eighty‐one percent of the participants had more than 5 years of experience in academia before COVID‐19 and therefore could reflect on their experience of turbulence when the study was conducted.

**TABLE 1:** Sample breakdown

Seniority	*N*	Types of schools or departments			
		Female	Male	Business and Management Schools	Other schools or departments (Health, Social Policy, Sociology, Sports Science, Psychology, Politics, Geography, Chemistry, Languages, Arts, Film & Media and Data Science)
Dean	2	2	0	2	0
Professor	22	10	12	21	1
Reader	2	2	0	0	2
Senior lecturer	29	14	15	19	10
Lecturer	30	13	17	19	11
Research and teaching fellows	17	10	7	10	7
**Total**	**102**	**51**	**51**	**71**	**31**

The majority of participants were from business and management schools (*n* = 71), and 31 were from other fields and types of schools (see Table 1). The challenges of working remotely were broadly similar across the schools sampled. A comparative study was beyond the scope of this paper, but some differences are worth noting. For instance, business schools often have a higher proportion of international students than other schools in UK universities. Indeed, the challenges of time zone differences and language barriers when interacting with students during online classes or one‐to‐one meetings were highlighted by a greater proportion of participants working in business schools. Participants in other schools faced different challenges, for instance when equipment was used during teaching (e.g., chemistry or film and media) or where work placements were an important part of a course (e.g., nursing, psychology).

The interviews were part of a broader project conducted in 2020 focussing on changing work arrangements and HRM in academia. Table 2 shows a monthly summary of the interviews. Out of the 102 participants, 10 were interviewed before 23/03/20 when the first national lockdown was imposed. At this point there were concerns regarding how COVID was circulating in the UK, many international students were unable to leave China and there had been discussions around how teaching delivery may need to change. These participants were then interviewed a second time after 23/03/20 (shown as ‘follow‐up interviews’ in Table 2) to gather their experiences of working from home during the lockdown, a topic that was not discussed in their first interview. In addition to the 10 follow‐up interviews, 11 of the participants were contacted again during the analysis stage to confirm a point made in their initial interview and then clarify or expand on it. This was done by e‐mail (eight participants), or by asking a small number of follow‐up questions through Zoom (three participants). For instance, a participant stated in their initial interview that homeworking could be beneficial and another participant stated that homeworking deteriorated for them over time, but their experiences were not adequately probed in their initial interview.

**TABLE 2:** Monthly breakdown of the interviews conducted during 2020 with 102 participants

Month	Number
February	**7**
March	9
April	33
May	22
June	27 (1 was a follow‐up interview)
July	5 (2 were follow‐up interviews)
August	2
October	4 follow‐up interviews
November	2 follow‐up interviews
December	1 follow‐up interview

The following types of questions were posed in the interviews. How has homeworking during COVID impacted you? How often did you work from home previously? How have you experienced online teaching? How have you experienced online meetings? How would you describe your relationship with your line manager/colleagues/students during this period? Have you been balancing caring responsibilities with work? How have you experienced not being able to meet wider family and friends in‐person? Have you adopted any strategies to deal with the challenges you have faced? How has the university or its HR department offered support during this period? In line with a semi‐structured interview approach, these types of questions were supplemented with questions to probe the participants' responses accordingly.

The interviews lasted an average of 63 minutes and were conducted mainly online or by phone because of the need for physical distancing. The interviews were digitally recorded, and the data obtained were fully transcribed and checked for accuracy before being analysed.

### 4.2 Data analysis

A thematic template‐based approach was used to systematically analyse the participants' perspectives because of its flexibility to oscillate between the interview data and the literature (King, 2012). We created a preliminary template to code four transcripts and then asked third‐party colleagues to code the anonymised interview transcripts without the preliminary template. This process enabled us to check the initial data coding and minimise the potential for confirmatory biases to affect the coding. We then discussed potential differences in the coding and made minor adjustments where needed.

**FIGURE 1:**
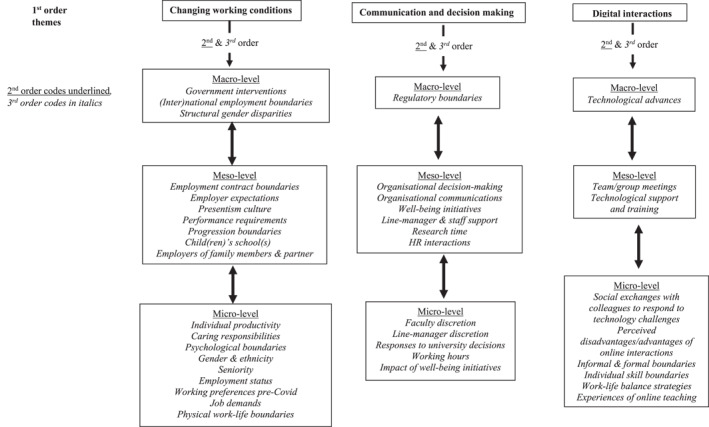
Interview and documentary data coding structure

NVivo12 was used to complete the coding and analysis. The authors reviewed, discussed, and refined the coding structure and node labels of the first five transcripts. After coding 20 transcripts, the authors rechecked and discussed the coding in detail. This progressive and iterative review process helped minimise the potential for the reliability and rigour of our coding to wane over time (coding drift) and continued until all the transcripts had been fully coded. Figure 1 sets out the coding structure.

As indicated in the Findings section, the coding process suggested that gender influenced the participants' responses with regard to caring responsibilities. Additionally, 31% of the respondents were non‐UK nationals and therefore more likely to have family outside the UK, which generated different challenges for them during COVID‐19. A limitation of the study is that 88% of the participants were white. The participants discussed the pre‐existing progression challenges BAME academics face, but more BAME participants could have provided an insight into how ethnicity influenced faculty experiences. The next section reveals the participants' experiences of homeworking on an enforced basis.

## 5 FINDINGS

The findings are divided into three thematic sections examining the respondents' experiences of homeworking during the turbulence caused by COVID‐19 and enforced lockdowns. The data elucidates how experiences of macroturbulence were shaped by social exchange relationships and different boundaries at multiple levels.

### 5.1 Enforced homeworking under rapidly changing scenarios

Twenty‐six of the participants reported that the time and cost savings from not commuting to work were significant positive spill over effects of working from home. Some of those who worked in their university office regularly before COVID‐19 stated that micro‐level social relationships and exchanges with colleagues or line managers often made it difficult to maintain existing time boundaries, and so increased their workloads and prolonged their working time.

If you're in your office, you're more likely to be asked to do something extra, either from a colleague or manager and you want to help them out but considering everything else we have to do … and it tends to be the same people who pick up the extra stuff.

(P13, Professor)

Furthermore, in some universities, a meso‐level presenteeism culture and/or heavy teaching loads placed pressure on staff to regularly work onsite before COVID‐19, generating tensions over personal and professional discretion and work‐life balance. Homeworking changed the nature of these tensions by reconfiguring physical work‐life boundaries and could provide staff with greater discretion over how they spent their time.

Sixty‐two of the participants regularly worked from home before the crisis, which potentially made the transition to homeworking easier. However, a key theme in the interview data was how the spill over effects of working at home and micro level personal control over boundaries to the temporality of work depended on individual circumstances and working environments.

Macro‐level regulatory boundaries prevented individuals from leaving their home to engage in physically proximate social relationships and exchanges with wider family and friends, some of whom were living outside the UK. COVID‐19 also prompted the UK government to suddenly close schools as part of the national lockdown and advise vulnerable individuals to protect themselves. Participants discussed the challenges of balancing expectations within social exchange relationships with their employer and those with individuals who they were caring for (29 participants) and/or those with their partner, wife, husband, wider family, and friends (50 participants). The spill over effects from COVID‐19 and homeworking generated tensions and threw work‐life boundaries into a new state of flux.

Tensions within work or family relationships can easily arise … Academics are in survival mode. Even getting food for themselves, their partners their families is a challenge. Everyone has different responsibilities outside work that they're trying to balance.

(P46, Professor)

Working from home is nothing that new. What's new and problematic is that I've got three young children who are not at school or nursery, and they've gone feral. They're down here every few minutes … I'm probably 30% of what I should be productivity‐wise.

(P31, Senior Lecturer)

The physical and psychological space the participants had to do their work at a micro‐level was affected by the size and layout of their home and how the available space(s) were being used for multiple purposes. In some cases, a spill over effect was the blurring of the boundaries between the work and occupations of different household members, because family members or partners were suddenly working from home at the same time.

I'm quite used to homeworking, but it's completely different when there are three of us now homeworking, and we can't go anywhere else. We have different jobs, but we all need to make calls, attend online meetings, use the Wi‐Fi constantly. Some rooms are better for Wi‐Fi than others, so we're having to work out who's working where.

(P41, Senior Lecturer)

Even if you've finished work for the day, you're then eating your dinner in the same place that you were working all day, so that kind of blurring happens not just in terms of the activities that you're doing but your sense of where you are.

(P8, Lecturer)

The physical and psychological segregation of work and life improved over time for some, as they were able to set up home offices or became more accustomed to their new work‐life arrangements. Twenty‐one of the participants reported experiencing severe anxiety, which affected their productivity, but for most of the participants, this anxiety dissipated over time. For other members of the sample, homeworking was becoming more rather than less problematic.

Initially colleagues were very positive, but not so much now. I think some of that is to do with people's mental health … at home … my e‐mail is [full up with messages from] very tired, frustrated, and annoyed colleagues.

(P4, Senior Lecturer)

About 22% of the male participants struggled to balance their work and caring responsibilities compared with 35% of the female participants. Most of the participants acknowledged that their female colleagues tended to encounter more difficulties than their male colleagues in reconciling social exchanges with family members and the demands of their job during the crisis. This could spill over and affect their progression through meso‐level professional seniority boundaries and exacerbate pre‐existing structural gender disparities over the short‐ and long‐term.

I think we've seen … a gender difference playing out where women are finding it much less straightforward to say, ‘Well, I'm going to prioritise research within my time’. And we're seeing this to an extreme at the moment, where I'm hearing predominantly from male colleagues that they have never been able to do as much research. And then female colleagues just pulling their hair out about the teaching and assessments they've got at the moment on top of caring responsibilities.

(P45, Dean)

Pre‐existing tensions around the challenges of progressing through meso‐level seniority boundaries and meeting performance requirements triggered concerns particularly among more junior staff that they needed to move forward despite the disruption they were facing. In addition to gender, 30% of the BAME participants discussed how these pre‐existing tensions and progression challenges could be influenced by ethnicity. A small number (*n* = 2) of the total sample (*n* = 102) stated that they had had discussions on how the spill over effects of the crisis would be considered when it came to their career progression to support staff within changing social exchange structures and relationships.

The crisis also exposed pre‐existing tensions around macro‐level casualisation. Fifty‐three percent of those on temporary contracts were nearing the end of their contracts. The following quote exemplifies how a research assistant sought to apply micro‐level strategies to manage employment boundaries and balance social relations and exchanges at work and home.

You're always a parent and always a worker the whole time. It's so overwhelming … but you've got to keep going. My husband has been working Monday, Friday and Wednesday afternoons, and I’ve been working Tuesday, Thursday and Wednesday mornings, but I'm working 8:00 a.m. to 10:00 p.m. Last night, I worked on a job application … until midnight.

(P30, Research Assistant)

This thematic section has focussed on how the participants experienced change and uncertainty in different ways during the turbulence of the COVID‐19 crisis. The next examines how the universities responded to this unfolding context for work and HRM.

### 5.2 Communication and decision‐making during the pandemic and lockdowns

Macro‐level government interventions meant that some decisions regarding university operations were outside a university's boundaries of control. Nevertheless, a range of communication approaches were adopted, as exemplified by the following account:

I would say the university is doing quite well … There's constant communication, and it's quite open. Although it's quite scary, they have been very transparent in terms of the financial situation. The communication contrasts significantly with my previous university.

(P20, Senior Lecturer)

Fifty‐one of the participants stated that their micro‐level control boundaries were affected by top–down communications, as illustrated in the following expert. This was perceived as an unfair social exchange with their employer and spilt over to affect social relations and exchanges between different parties as information was cascaded hierarchically.

Decisions have been made centrally, which then get reinterpreted at school level. And then they get reinterpreted at division level, so there's been so much confusion … there have been a number of things that have come up, mostly around teaching, when students have asked questions. But also around things like leave … and stuff like that. No one is quite sure what's going on, and … it depends who you ask, what the answer is.

(Professor, P11)

The relative clarity of university communications could spill over to shape temporary staff perceptions of the longevity of exchange relationships with their employer. A senior lecturer who was a programme director (P40) explained how he engaged in social exchanges by reassuring those who felt their university jobs were already fragile:

I had to have a conversation with my two youngest members of staff to reassure them because of a not‐very‐well‐worded university disclosure. If you read it at first glance without knowing any [details], it basically said we're going to follow with redundancies, which is not the case at all.

Most of the participants stated that their universities sent communications advising staff to focus on their welfare while working from home, which could be interpreted as an attempt by universities to support perceptions of reciprocal social exchanges. Some identified a shift in meso‐level university expectations and control strategies following resistance from colleagues because of their perceptions of continuing and/or increasingly disparate social exchanges.

At first, they said, ‘We expect staff to work diligently and deliver’, but then the university were basically inundated with e‐mails saying, ‘How do you expect that to happen?’ It's only the government that can reopen schools and nurseries. Some people might have time on their hands but not everyone. Then they sent an e‐mail recognising that we cannot work to full capacity and saying they just expect us to do what we can.

(P6, Senior Lecturer)

Well‐being days were also introduced at some of the participants' universities, including additional leave or specific days in the week without e‐mails and meetings, which provided some with relief and the scope to use these sanctioned temporal boundaries to manage the conflicting pressures they were facing. However, many stressed that these meso‐level well‐being initiatives were undermined by the volume of work they were simultaneously expected to complete and the demands of different internal and external parties, with limited meso‐level support and resources in place.

There's a difference between the rhetoric and what I feel the real expectation is … I haven't stopped. I've had loads of deadlines. I've been working weekend after weekend.

(P19, Lecturer)

We get these university communications about well‐being, but then you get another e‐mail from someone else with a deadline … or needing something and students e‐mailing you left, right and centre. So there's a disconnect.

(P28, Professor)

Furthermore, these well‐being communications were mainly sent from the leadership of their respective universities rather than their HR departments. Indeed, most reported that HR had effectively faded into the background and had been playing a nominal role in shaping the emotional and practical support available to staff within work‐centred exchange relationships.

For me, this would have been the perfect time for HR to really consolidate or elevate its position beyond this kind of bureaucratic or managerial role to really start to engage with people about how you are coping with all this stuff. Do you need support ideas? Do you need someone to talk to honestly about how you feel in yourself? How you're dealing with childcare? Home schooling? Whatever it is?

(P23, Senior Lecturer)

Sixty‐five of the participants discussed how line managers faced challenges navigating tensions within social exchange relationships, meso‐level university communications, decisions, and control boundaries. Ten of the participants were senior leaders at school‐level and mid‐level leaders at a broader university‐level (Deans, Associate Deans) or line managers (department/group heads) themselves. In some of the participants' universities, research time allocations were curtailed to prioritise teaching. This included cancelling or postponing sabbaticals. A business school Dean explained how she had tried to resist reductions in research time allocations because giving research time ‘back’ to staff would be unrealistic in practice given the nature of academic work.

A line manager noted how prioritising teaching over research was likely to spill over to affect progression through seniority boundaries (P18, Professor). This was because this impacted on exchange relationships between academics and their employer, line manager and colleagues.

It's a complete nightmare for progression issues because we've been told that research isn't prioritised, teaching is the priority, and … we're not doing our annual performance reviews as a result. That means there's a chunk of people who would have been hoping for promotion … who are probably not going to have any mechanism to do that.

As exemplified below, line manager approaches to influence social exchange relationships varied dramatically. Managers who were committed to supporting the wide‐ranging and changing needs of staff and students also needed support. However, individual managerial approaches could generate tensions and challenges.

My line manager does everything he can, and he has kids. It's middle managers at different levels that have to negotiate that messaging … it's terrible because it puts all the pressure on them. They need support.

(P34, Lecturer)

I genuinely feel like I’m in the middle of a swimming pool, struggling, and management are standing around the outside of the pool, congratulating me on how well I'm doing for not drowning and managing to just about get mouthfuls of air, and no one is swinging a chair in so that I can hang onto it. I'm surrounded by other colleagues who might be treading water, or they might be drowning like I am, or some of them seem to be floating about, smoking cigars, having quite a nice time.

(P9, Senior Lecturer)

The crisis reconfigured boundaries and social exchange relationships between colleagues and students by enforcing online communication. The next section reveals the tensions underpinning the participants' experiences of digital interactions during the crisis.

### 5.3 The shift towards exclusively digitalised interactions

Most participants highlighted the challenges of digital interactions during the crisis. A key issue revolved around micro‐level informal interactions, which played a major role in social exchanges among colleagues to share information and ideas but tended to be more difficult and time‐consuming online. Moreover, six of the recently appointed participants explained the challenges of interacting online when they had not yet met colleagues in‐person.

Some of the interviewees appreciated how opportunities to participate in online meetings and events had increased meeting attendance and could potentially help realign the boundaries between staff and university control over their work‐life balance in the future by reducing the need to travel to work as often, which may then spill over to support the perceived fairness of exchange relationships with their employer. Others were less enthusiastic about these boundary changes, emphasising the reduced visibility of body language indicators and the more draining nature of long and/or successive online meetings compared with interspersed in‐person meetings.

Furthermore, homeworking also exposed meso‐ and micro‐level tensions around meetings and digital communications, which pre‐dated the crisis and could create perceptions of inequitable social exchange relationships. Thirty‐six of the participants said that the number, length and structure of compulsory online/in‐person meetings organised by different individuals within universities needed to be re‐evaluated in the future, as they continued to cross temporal and locational work and control boundaries.

So we're in this global pandemic, and the first thing universities want to do is have lots of meetings again, virtual meetings rather than physical meetings, but there's no need for a lot of these meetings. It's different people trying to over‐control things.

(P50, Professor)

The participants explained how some line managers organised online meetings to communicate relevant information and identify staff needs. Others organised too few, which meant workers lacked support within the social exchange, or too many, which was perceived as a means of exercising control.

We've been having team meetings every week, which is more than we ever had before, and I'm finding them more frustrating because we would never normally see each other that much, and so it's more work again.

(P16, Senior Lecturer)

We didn't have a department meeting for the first [six] weeks. In the end, I organised something informal because people needed support.

(P9, Senior Lecturer)

Digital interactions could extend working hours for those with or without formal managerial responsibilities. This was due to differing temporal availability and academics seeking to balance social exchange relationships with multiple parties.

Some meetings, I've logged into in the middle of the night because I'm outside of the UK [because of caring responsibilities], but when they're unnecessary, I'll just turn my camera off and try to stay awake.

(P87, Professor)

You sort of feel a bit like you're chained to your computer or mobile phone. People [contact] you on your own personal mobile, and you're getting either WhatsApp messages or phone calls about work on a Sunday morning, and it's just a bit much. It's not always important stuff.

(P22, Senior Lecturer)

Many of the participants implemented micro‐level strategies to manage work‐life boundaries, which were in some cases developed with colleagues as part of social exchanges. Since the outbreak, some had set up out‐of‐office messages after 5 p.m. on weekdays and over weekends, made internal/external colleagues and students aware of their caring responsibilities in their e‐mail signatures, deleted e‐mail apps from their personal phones, set their status to ‘appear offline’ when using Microsoft Teams or avoiding signing in unless for scheduled meetings, turned their camera off in meetings, refrained from attending unnecessary meetings or used different technologies and software to segregate work and life (e.g., the use of a laptop for work and a tablet outside working hours).

Only seven participants had significant experience in online teaching before the enforced homeworking. Meso‐level technological infrastructure was highlighted as an important source of support within the social exchange with their employer but varied across institutions and exposed pre‐existing tensions.

You need robust platforms, and you need learning technologists. We haven't even got one learning technologist within the school. There are a few in the university, but we haven't got their services, so we're kind of scratching around.

(P49, Professor)

Our IT people at the moment are amazing … There are dedicated people to help us. But … those people in IT [whom] we're completely dependent on are going through a review at the moment to see if their service can be centralised, which means less staff.

(P10, Professor)

Many participants felt that universities needed to acknowledge micro‐level skill boundaries and that the provision of training to upskill staff may be required to support social exchange relationships. However, many did not have time to attend relevant training and use technology innovatively before COVID‐19, so the increased workload and turbulence they were facing further disrupted their temporal and work‐life boundaries.

I'm not particularly techy. I've come from a generation where there were no computers. I found moving to remote working quite challenging because it's very time‐consuming. There has been a reluctance by the institution prior to this to actually train people and give them an opportunity to make their lives more accessible to these technologies.

(P5, Senior Lecturer)

Where meso‐level technological support was lacking and university communications unclear, the participants explained how they established or re‐enforced informal networks and engaged in micro‐level social exchanges with colleagues. As one of the participants stated:

Everyone in the division [is] running around, thinking of really useful things to do, and people with five or ten minutes of experience on Zoom are passing on their vast knowledge to everyone else. We're all sharing our accounts and setting up meetings for other people. We're all helping each other teach online. I've stood in for a couple of colleagues who were ill with COVID‐19.

(P37, Professor)

However, such collegiality could make it difficult to preserve micro‐level research time boundaries and extend working hours. This is exemplified by the following participant's account:

What I'm doing now … will hopefully help colleagues. But then I do get resentful when I know people are just keeping their heads down and writing lots of papers and I’m not.

(P49, Professor)

This section has shed light on how enforced homeworking and the macroturbulence generated by COVID‐19 affected HRM, working conditions and individual experiences in disparate ways. These dialectics and spill over effects were steered by the nature of social exchange relationships between multiple parties and a range of boundaries spanning different levels.

## 6 DISCUSSION AND CONCLUSIONS

**FIGURE 2:**
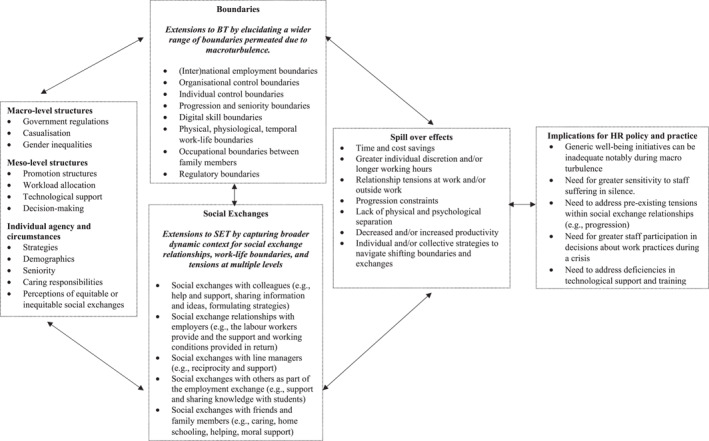
Theorising and analysing experiences of macroturbulence on work and HRM by fusing boundary and social exchange theory

The findings from this study identify three main contributions to extend the theorisation of the impact of macroturbulence on worker experiences and HRM. First, the paper proposes and empirically applies a relational multilevel framework integrating SET and BT. Boundary theory can be applied to examine changing physical, temporal, and psychological work‐life boundaries (Delanoeije et al., 2019). However, it lacks a focus on the nature of social exchange relationships and individual perceptions of obligations and expectations, which SET provides (Blau, 1964; Cropanzano et al., 2017; Prouska et al., 2022). As shown in Figure 2, we identify a range of social exchanges and boundaries that cut across the work‐life interface at different levels. Perforated lines are used in the figure to reflect permeable social exchanges between boundaries and levels. We argue that how these boundaries and exchanges interact and generate spill over effects should be an important focus for future research.

Second, as Figure 2 shows, our framework explains the complex interplay between structures and agentic behaviours, which are usually overlooked in empirical HRM research applying SET or BT in the context of macroturbulence as well as more broadly (Cross & Dundon, 2019; Harney, 2019). The findings revealed how staff experiences of working remotely during the pandemic were shaped by tensions relating to macro‐level structures such as government regulations, gender inequalities, casualisation and meso‐level organisational structures connected to progression, workload allocation, technological support, and decision‐making. The findings also capture agency dynamics within these structures, such as how individuals applied strategies to navigate or counter the realignment of exchange relationships or the infringement of work‐life boundaries.

Relatedly, the paper probes the intricacies underpinning the boundaries of organisation and staff control. During the pandemic, the boundaries of a university's control were constrained by regulatory structures and the intensification of pre‐existing economic conditions (Thompson, 2013), but most of the participants felt that universities had the discretion to shape their communication strategies and the nature of decision‐making. Top‐down decision‐making created perceptions of less equitable social exchanges. The way line managers used their agency to filter and navigate university communications and decisions and the level of support they received within segmented organisational structures varied and added to the complexity of fostering positive social exchange relationships with staff. Moreover, although opportunities to engage in online rather than in‐person interactions at work could potentially enhance the quality of social exchange relationships between peers and managers by extending individual control over work‐life boundaries, we capture how staff experiences are far more complex when homeworking is enforced (Anderson & Kelliher, 2020).

Third, the paper sheds much‐needed light on the uneven impact of a macro crisis at the meso and micro levels. This contributes new insights into research identifying the significance of individual circumstances in the context of an economic crisis (e.g., Chatrakul Na Ayudhya et al., 2019) and into the growing literature challenging the notion and effectiveness of universal best‐practice HR (Cafferkey et al., 2019; Harney, 2019). As shown in Figure 2, staff experiences during the pandemic stemmed from the confluence between individual circumstances and meso‐ and macro‐level factors, including the demands of exchange relationships with numerous parties, such as internal and external colleagues, line managers, students, partners, family members and other members of society. Importantly, colleagues helped others without any guarantee their support would be reciprocated in the future. COVID‐related deaths rose rapidly, and some participants referred to colleagues or relatives they had lost or had suffered from COVID‐19. Therefore, the concept of reciprocity embedded within SET is more nuanced than typically presented in the literature, particularly in turbulent crises when greater social exchange is needed. Tensions between social exchange relationships and boundaries can produce micro‐level spill over effects that are not always obvious to colleagues, line managers or employers, especially if staff remain silent (Prouska & Psychogios, 2018). For instance, some participants lived alone and had physical and psychological space to work but worked evenings and weekends because of social exchange relationships and progression boundaries, thereby affecting their well‐being. The findings also highlight the need to examine how macroturbulence impacts temporary workers, who are often neglected in research applying SET or BT.

The findings generate implications for work and HRM in the higher‐education sector, which are likely to resonate more widely. Notably, during macroturbulence, generic well‐being initiatives are unlikely to address individual worker and line manager needs when they seek to manage exchange relationships and boundaries, particularly when they may be suffering in silence. Importantly, addressing pre‐existing tensions within social exchange relationships between staff and their employer, such as around progression structures, is likely to shape staff experiences of future turbulence. Moreover, individual agency needs further consideration particularly regarding the agency employers have despite structural constraints to provide greater staff participation in decisions about work practices during a crisis, along with staff agency to resist inequitable exchange relationships individually or collectively. Finally, if organisations further incorporate technology into working practices in the aftermath of COVID‐19, greater employer awareness of deficiencies in technological support and training must be raised, including how the extent of digital interactions shapes staff experiences and productivity as well as the perceived necessity of interactions at different points in time.

The main limitations of the study were the exclusive focus on academics and the size and nature of the sample, which was uneven across schools. The potential biases of the researchers, who were homeworking during the outbreak, are acknowledged. However, we sought to minimise these limitations by building a large sample of participants with different views and asking third‐party researchers to review our interpretation of the data.

Future research could adopt single‐ and multi‐case study approaches to identify similarities and variances in the impact of such turbulence and homeworking on HRM across occupations, industries, and countries. Analysing how the nature of social exchange relationships interact with multiple boundaries to shape worker experiences of HRM during other crises is also important to better understand the multilevel impacts of subsequent turbulence.

## CONFLICT OF INTEREST

No conflict of interest in producing this paper and there was no funding.

## Data Availability

The data are unavailable due to privacy/ethical restrictions.
